# Asiatic Acid Inhibits Pro-Angiogenic Effects of VEGF and Human Gliomas in Endothelial Cell Culture Models

**DOI:** 10.1371/journal.pone.0022745

**Published:** 2011-08-03

**Authors:** Chandagirikoppal V. Kavitha, Chapla Agarwal, Rajesh Agarwal, Gagan Deep

**Affiliations:** 1 Department of Pharmaceutical Sciences, School of Pharmacy, University of Colorado Denver, Denver, Colorado, United States of America; 2 University of Colorado Cancer Center, Aurora, Colorado, United States of America; Roswell Park Cancer Institute, United States of America

## Abstract

Malignant gliomas are one of the most devastating and incurable tumors. Sustained excessive angiogenesis by glioma cells is the major reason for their uncontrolled growth and resistance toward conventional therapies resulting in high mortality. Therefore, targeting angiogenesis should be a logical strategy to prevent or control glioma cell growth. Earlier studies have shown that Asiatic Acid (AsA), a pentacyclic triterpenoid, is effective against glioma and other cancer cells; however, its efficacy against angiogenesis remains unknown. In the present study, we examined the anti-angiogenic efficacy of AsA using human umbilical vein endothelial cells (HUVEC) and human brain microvascular endothelial cells (HBMEC). Our results showed that AsA (5–20 µM) inhibits HUVEC growth and induces apoptotic cell death by activating caspases (3 and 9) and modulating the expression of apoptosis regulators Bad, survivin and pAkt-ser473. Further, AsA showed a dose-dependent inhibition of HUVEC migration, invasion and capillary tube formation, and disintegrated preformed capillary network. AsA also inhibited the VEGF-stimulated growth and capillary tube formation by HUVEC and HBMEC. Next, we analyzed the angiogenic potential of conditioned media collected from human glioma LN18 and U87-MG cells treated with either DMSO (control conditioned media, CCM) or AsA 20 µM (AsA20 conditioned media, AsA20CM). CCM from glioma cells significantly enhanced the capillary tube formation in both HUVEC and HBMEC, while capillary tube formation in both endothelial cell lines was greatly compromised in the presence of AsA20CM. Consistent with these results, VEGF expression was lesser in AsA20CM compared to CCM, and indeed AsA strongly inhibited VEGF level (both cellular and secreted) in glioma cells. AsA also showed dose-dependent anti-angiogenic efficacy in Matrigel plug assay, and inhibited the glioma cells potential to attract HUVEC/HBMEC. Overall, the present study clearly showed the strong anti-angiogenic potential of AsA and suggests its usefulness against malignant gliomas.

## Introduction

Neo-angiogenesis refers to the formation of new blood vessels from existing parent vessels and is considered crucial for the transition of tumors from a dormant to malignant state [1], [2]. Angiogenesis is now established as one of the hallmarks of cancer, and it is estimated to be responsible for over 90% of all cancer deaths [3]. For example, malignant gliomas are considered incurable largely due to sustained and excessive angiogenesis, and approximately 77% of glioma patients die within the first year of their diagnosis [4], [5], [6], [7], [8], [9], [10]. Infact, gliomas are among the most vascularized human tumors, and excessive vasculature is induced by several pro-angiogenic factors produced by glioma cells [11], [12]. Hence, one possible treatment strategy that may improve glioma patient's outcome is the use of angiogenesis-targeting agents.

Vascular endothelial growth factor (VEGF), a diffusible glycoprotein, is a widely over-expressed pro-angiogenic factor in most solid cancers and plays a critical role in various steps involved in angiogenesis including endothelial cell proliferation, migration and tube formation [13], [14]. VEGF secreted by tumor cells interacts with VEGF receptors (VEGFRs) in endothelial cells and stimulates downstream signaling molecules such as mitogen-activated protein kinases (MAPKs) and Akt to promote the growth, survival and migration of endothelial cells [15], [16], [17]. Therefore, inhibition of VEGF secretion by tumor cells as well as VEGF regulated signaling in endothelial cells could be important in targeting tumor angiogenesis. The U.S. Food and Drug Administration (FDA) has recently approved Avastin, an antibody against the VEGF receptor, for the treatment of various cancers [18], [19]. Avastin has shown promising pre-clinical and clinical activity against metastatic colorectal cancer in combination with fluorouracil [20], [21]. In addition, it was also recently approved for the treatment of recurrent gliomas [22], [23]. However, the randomized phase II BRAIN study showed that the median survival rate after Avastin treatment is limited and it was only 2–3 months longer compared to other treatments [24]. Moreover, Avastin use is costly and could cause serious side effects such as gastrointestinal perforation and bleeding. Alternatively, we suggest the use of agents that are small, non-toxic, safe, affordable, and efficacious in inhibiting angiogenesis in gliomas and other cancers. In this direction, present study examines the anti-angiogenic efficacy of one such small molecule namely Asiatic Acid (AsA).

AsA is a pentacyclic triterpenoid derived from the tropical medicinal plant *Centella asiatica* (family-Apiaceae). Beneficial effects of AsA have been reported in wound healing, UV-induced photoaging, glutamate- or β-amyloid-induced neurotoxicity and hepatofibrosis [25]. Furthermore, there are numerous reports suggesting the strong neuroprotective and anti-cancer efficacy of AsA [26], [27], [28], [29], [30]. For example, AsA treatment has been reported to induce apoptotic death in human hepatoma and malignant glioma cells through enhancing the intracellular calcium release [31], [32]. In another study, Park *et al.* reported that AsA induces apoptosis in melanoma cells through increasing the levels of reactive oxygen species [33]. AsA as well as *Centella asiatica* extract have also been shown to possess strong efficacy against colon cancer cells [34], [35]. Despite these widely described anti-cancer properties, AsA has not been tested for its anti-angiogenic potential. In the present study, for the first time, we investigated the anti-angiogenic efficacy of AsA using human umbilical vein endothelial cells (HUVEC) and human brain microvascular endothelial cells (HBMEC). We also examined AsA activity in inhibiting the pro-angiogenic effects of VEGF as well as the conditioned medium from human glioma cells (LN18 and U87-MG) using HUVEC and HBMEC. Our results clearly showed that AsA moderately inhibits endothelial cell growth but strongly induces apoptosis as well as inhibits VEGF- and glioma conditioned media-induced tube formation and invasiveness in endothelial cells.

## Results

### AsA inhibits growth and induces apoptotic cell death in HUVEC

**Figure 1:**
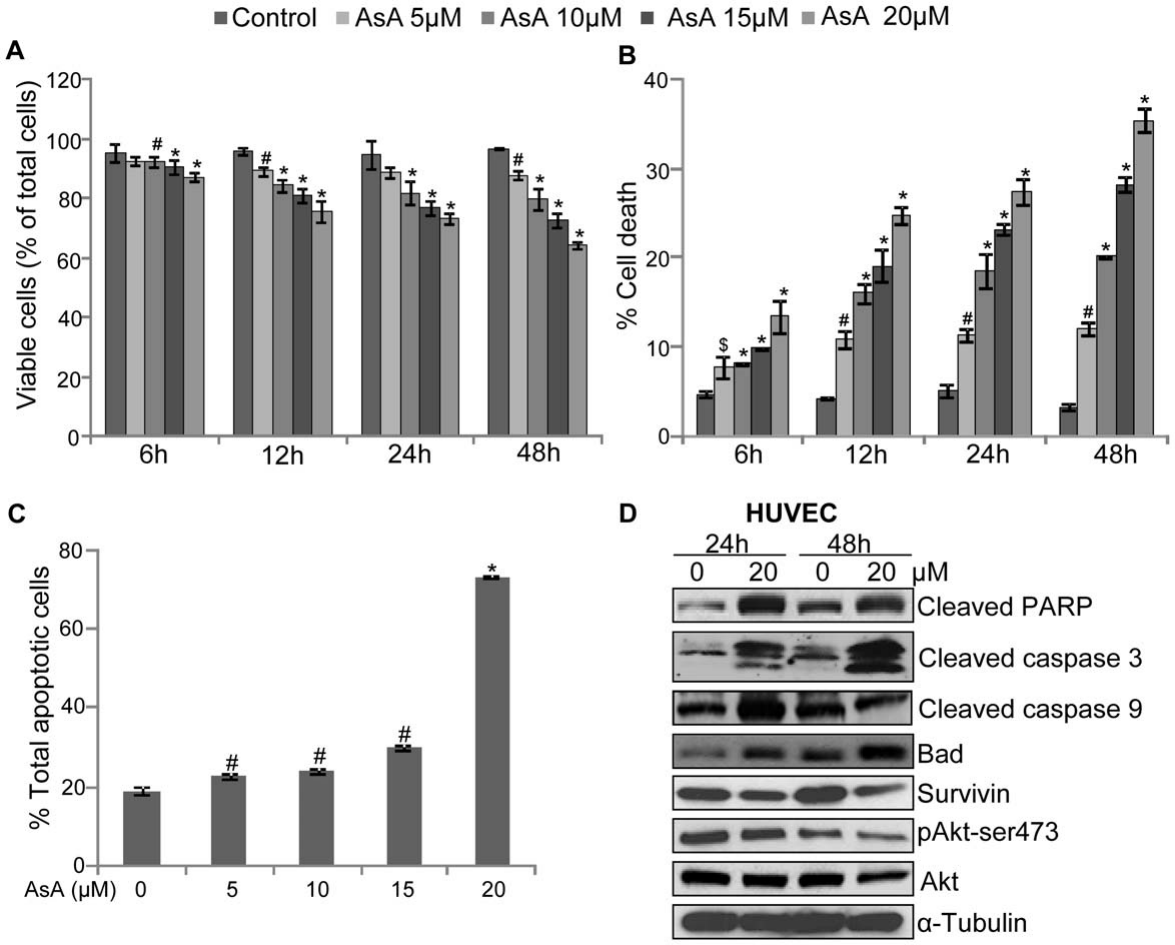
AsA inhibits growth and induces apoptosis in HUVEC. **A & B.** HUVEC (4×10^4^ cells per well) were treated with DMSO or different doses of AsA (5–20 µM) in complete HUVEC media for 6, 12, 24 and 48 h. At each treatment time, both adherent and non-adherent cells were collected and processed for the determination of viable cell number and dead cells percentage as mentioned in ‘Materials and Methods’. **C.** HUVEC were treated with DMSO or AsA (5–20 µM) for 24 h and analyzed for apoptotic cell population using annexin V/PI staining as detailed in ‘Materials and Methods’. In panels A, B, & C, each bar represents the mean ± standard deviation of three samples. These results were almost similar in two independent experiments. *, p≤0.001; #, p≤0.01; $, p≤0.05. **D.** HUVEC were treated with DMSO or AsA (20 µM) for 24 and 48 h. After each treatment time, total cell lysates were prepared and analyzed for cleaved PARP, cleaved caspase 3, cleaved caspase 9, Bad, survivin, pAkt-ser473, and total Akt by Western blotting as detailed in ‘Materials and Methods’. In each case, membrane was also stripped and reprobed with anti-α-tubulin antibody to confirm equal protein loading.

To determine the effect of AsA on endothelial cells, in the first experiment HUVEC were treated with various doses of AsA (at 5, 10, 15 and 20 µM), and its effect on cell number and cell death was analyzed as a function of time (6, 12, 24, and 48 h) by trypan blue exclusion assay. As shown in figure 1A, AsA treatment (5–20 µM) decreased the viable cell number (% of total cell number) by 7–13% (p≤0.01−0.001), 11–24% (p≤0.01−0.001), 11–27% (p≤0.001) and 10–35% (p≤0.01−0.001) after 6, 12, 24 and 48 h of treatment, respectively. AsA treatment also resulted in a dose-dependent increase in cell death. As shown in figure 1B, compared to DMSO control, cell death with AsA treatment (5–20 µM) was increased by 3–9% (p≤0.05–0.001), 6–20% (p≤0.01–0.001), 6–22% (p≤0.01–0.001), and 9–32% (p≤0.01–0.001) after 6, 12, 24 and 48 h, respectively. Next, apoptotic cell percentage in the total cell population was quantified by annexin V/PI dual staining. Results showed that AsA induces apoptosis in HUVEC in a dose-dependent manner especially at the highest AsA dose used i.e. 20 µM (Figure 1C). Accordingly, we used 20 µM dose of AsA to further analyze its effect on apoptosis-related signaling molecules in HUVEC. As shown in figure 1D, AsA treatment for 24 and 48 h resulted in PARP cleavage, which is a well established biomarker for apoptotic death [36]. AsA also increased the expression of cleaved caspase 3 and Bad after 24 and 48 h of treatment, while increase in cleaved caspase 9 level was observed only after 24 h of AsA treatment. Likewise, AsA also decreased the levels of anti-apoptotic and pro-survival molecules namely survivin and phosphorylated Akt-ser473 after 24 and 48 h of treatment (Figure 1D). Overall, these results clearly showed that AsA treatment decreases viability and induces apoptosis in HUVEC through altering the expression of pro-apoptotic and anti-apoptotic molecules.

### AsA inhibits motility and capillary structure formation in HUVEC

**Figure 2:**
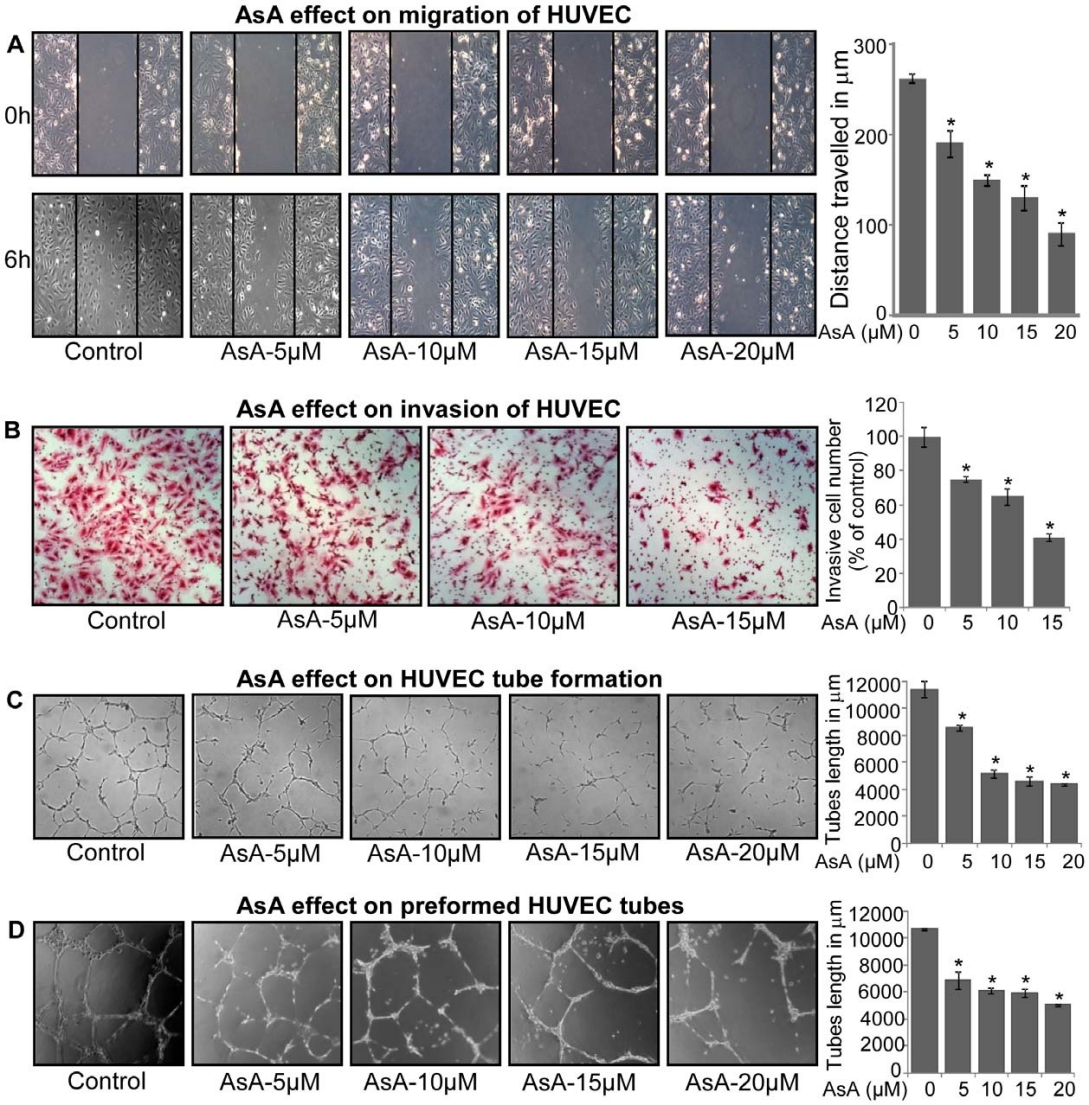
AsA inhibits motility and capillary-structure formation in HUVEC. **A.** Effect of AsA treatment on the migratory potential of HUVEC was analyzed through wound healing assay. Representative photomicrographs of initial and final wounds are shown at 100x magnification and migration distance was measured as detailed in ‘Materials and Methods’. Cell migration distance data shown are mean ± standard deviation of three samples for each treatment. **B.** Effect of AsA treatment on the invasive potential of HUVEC was examined using invasion chambers as detailed in ‘Materials and Methods’. Cell invasion data shown are mean ± standard deviation of three samples for each treatment. **C.** Effect of AsA on the tube formation of HUVEC was examined by plating HUVEC on the matrigel. After 6 h, tubular structures were photographed at 100x magnification and tube length was measured as described in ‘Materials and Methods’. Tube length data is presented as mean ± standard deviation of three samples for each treatment. **D.** Effect of AsA on the pre-formed tubes in HUVEC was analyzed and tube length was measured as detailed in ‘Materials and Methods’. Tube length data shown are mean ± standard deviation of three samples for each treatment. These results (A–D) were similar in 2–3 independent experiments. *, p≤0.001.

Endothelial cell motility i.e. migration/invasion is essential for the formation of new blood vessels during neo-angiogenesis, making it a critical event for tumor growth [37]. Accordingly, next we studied the effect of AsA treatment on the migratory and invasive properties of endothelial cells using wound-healing and transwell assays, respectively (Figure 2A & 2B). As shown in figure 2A, AsA treatment for 6 h inhibited the distance migrated by HUVEC approximately by 28% (p≤0.001), 43% (p≤0.001), 51% (p≤0.001) and 66% (p≤0.001) at 5, 10, 15, and 20 µM doses, respectively. Similarly, AsA treatment for 10 h inhibited the number of HUVEC invaded through the matrigel by 25% (p≤0.001), 35% (p≤0.001) and 59% (p≤0.001) at 5, 10 and 15 µM doses, respectively (Figure 2B). For these studies, we selected early time-points where we observed the least cytotoxicity of AsA towards HUVEC (Figure 1A & 1B).

Another important step during neo-angiogenesis is the formation and merging of tubes by endothelial cells forming a complex network of vessels and capillaries [38], [39]. To understand AsA effect on this biological event, we used two-dimensional matrigel assay and examined AsA effect on tubular structure formation by HUVEC. As shown in figure 2C, HUVEC plated on matrigel formed a massive network of tubes after 6 h (i.e. DMSO treated controls), which was disrupted by AsA treatment (5–20 µM) (Figure 2C). We also measured the average tube length in each case to quantify the inhibitory effect of AsA on the formation of tubular network by HUVEC. As shown in the bar diagram, AsA treatment inhibited the tube length approximately by 25% (p≤0.001), 55% (p≤0.001), 60% (p≤0.001) and 62% (p≤0.001) at 5, 10, 15, and 20 µM doses, respectively (Figure 2C). In another related experiment, we examined the effect of AsA treatment on preformed tubes by HUVEC, where we added AsA (5–20 µM) after HUVEC had already formed the tubular network on matrigel (after 10 h). In this experimental condition too, AsA treatment significantly disrupted the tubular network formed by HUVEC (Figure 2D). Quantification of tube length in this assay showed that AsA treatment inhibits the tube length by 36% (p≤0.001), 43% (p≤0.001), 45% (p≤0.001) and 53% (p≤0.001) at 5, 10, 15, and 20 µM doses, respectively (Figure 2D).These results clearly showed that AsA treatment inhibits motility and tubular structure formation as well as disrupts preformed capillary tubes by HUVEC.

### AsA inhibits VEGF-stimulated growth and capillary structure formation in HUVEC

**Figure 3:**
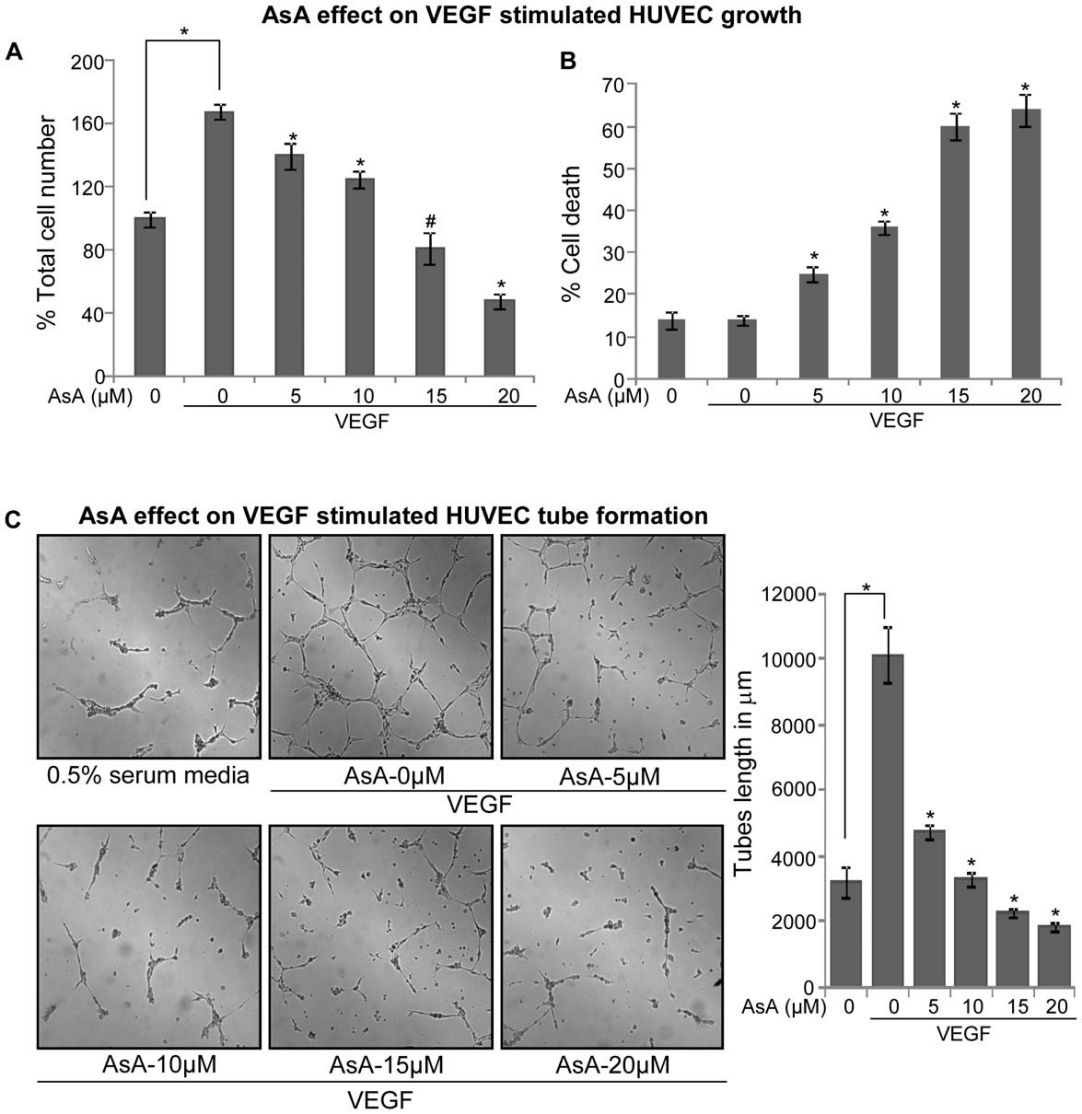
AsA inhibits VEGF-stimulated cell growth and capillary tube formation in HUVEC. HUVEC were grown under 0.5% serum conditions and treated with or without VEGF (10 ng/mL) and various doses of AsA (5–20 µM) for 12 h as described in Materials and Methods. After 12 h, both adherent and non-adherent cells were collected and processed for determination of total cell number (**A**) and dead cells percentage (**B**) as mentioned in ‘Materials and Methods’. **C.** HUVEC with or without VEGF (10 ng/mL) in 0.5% serum media were placed in 24-well plates coated with Matrigel and treated with AsA at indicated doses. After 10 h tubular structures were photographed at 100x magnification and tube length was measured as detailed in ‘Materials and Methods’. These results (A–C) were similar in 2–3 independent experiments. Each bar is representative of mean ± standard deviation of three samples for each treatment. *, p≤0.001; #, p≤0.01.

VEGF is the most important pro-angiogenic factor which is known to enhance proliferation, survival, and tube formation by endothelial cells [13]. Therefore, we next studied the effect of AsA on VEGF-stimulated cell growth and cell death in HUVEC. In this study, HUVEC grown under 0.5% serum conditions were treated with 10 ng/ml VEGF and various concentrations of AsA (5–20 µM). Cell number and cell death were analyzed by trypan blue exclusion assay after 12 h of AsA treatment. As shown in figure 3A, VEGF addition significantly enhanced the HUVEC growth (p≤0.001), which was inhibited by AsA in a dose-dependent manner. In comparison to VEGF alone, AsA at 5, 10, 15 and 20 µM doses decreased the VEGF-stimulated HUVEC number by 17% (p≤0.001), 26% (p≤0.001), 52% (p≤0.01) and 72% (p≤0.001), respectively (Figure 3A). AsA treatment also resulted in a dose-dependent increase in HUVEC death. As shown in figure 3B, cell death with AsA treatment increased by 1.8 fold (p≤0.001), 2.6 fold (p≤0.001), 4.3 fold (p≤0.001) and 4.6 fold (p≤0.001) at 5, 10, 15 and 20 µM doses respectively.

Next, we investigated the effect of AsA treatment on VEGF-stimulated tube formation in HUVEC. As shown in figure 3C, VEGF presence significantly enhanced the tubular network formation by HUVEC compared to HUVEC plated on matrigel under 0.5% serum containing HUVEC media alone; however, AsA treatment strongly inhibited the VEGF-stimulated tubular network formation by HUVEC (Figure 3C), and tube length measurement showed that AsA treatment inhibited the tube length approximately by 54% (p≤0.001), 68% (p≤0.001), 78% (p≤0.001) and 82% (p≤0.001) at 5, 10, 15 and 20 µM doses, respectively (Figure 3C). These results clearly showed that AsA strongly targets VEGF-stimulated cell proliferation and tubular structure formation in HUVEC.

### AsA inhibits VEGF-stimulated growth and capillary structure formation in HBMEC

**Figure 4:**
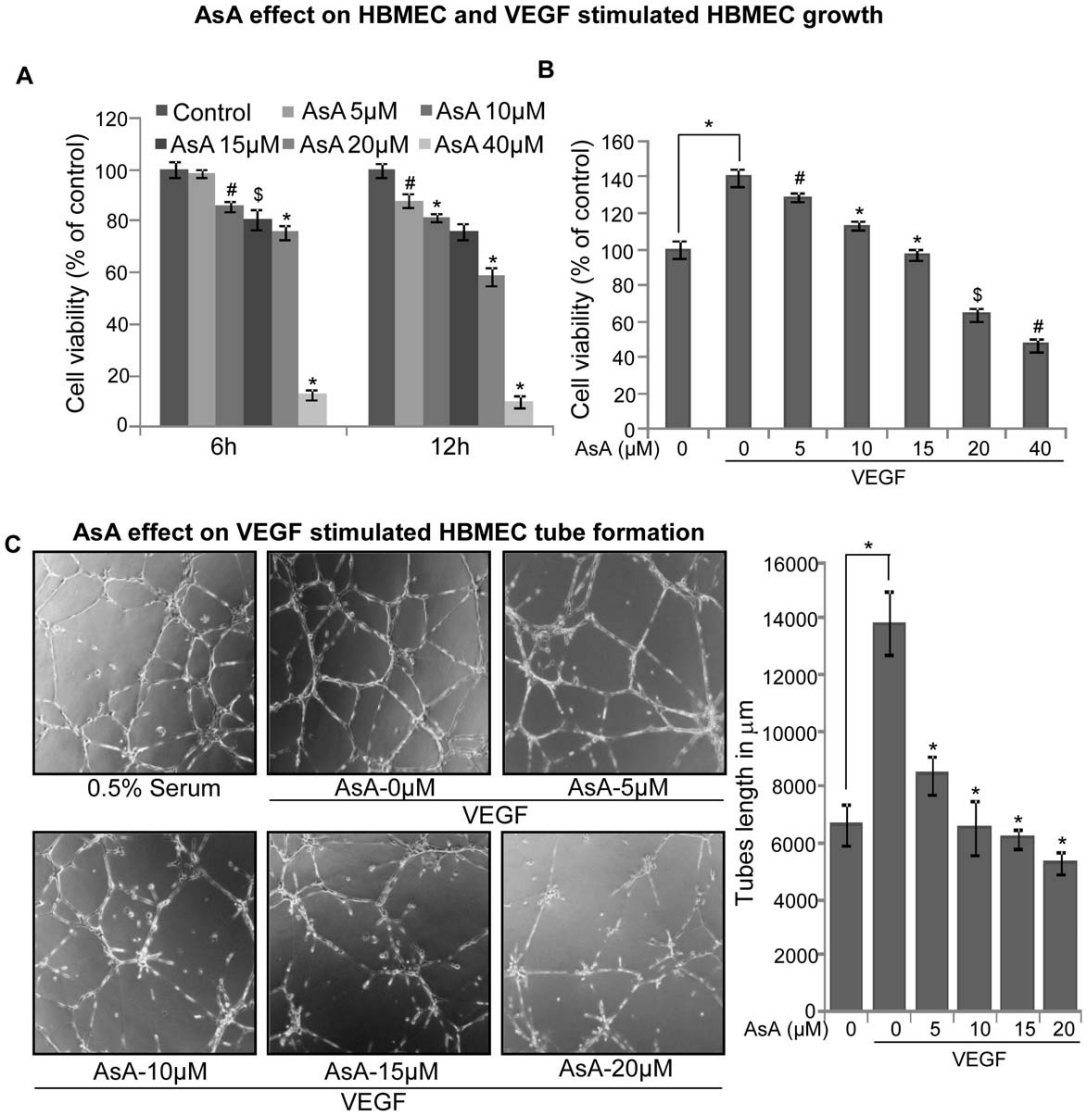
AsA inhibits growth and capillary tube formation in HBMEC. **A.** HBMEC (1×10^3^ per well) were seeded in a 96-well plates in complete media and next day treated with DMSO or AsA (5–40 µM) and cell viability was determined at 6 and 12 h after AsA treatment through MTT assay. **B.** HBMEC were grown under 0.5% serum conditions and treated with or without VEGF (10 ng/mL) and various doses of AsA (5–40 µM) for 12 h. The cell viability was determined by MTT assay. **C.** HBMEC (4×10^4^ per well) with or without VEGF (10 ng/mL) in 0.5% serum media were placed in 24-well plates coated with Matrigel and treated with AsA at indicated doses. After 10 h, tubular structures were photographed at 100x magnification and tube length was measured as described in ‘Materials and Methods’. Tube length data is presented as mean ± standard deviation of three samples for each treatment. *, p≤0.001; #, p≤0.01; $, p≤0.05.

In order to rule out that the observed effect of AsA on VEGF-stimulated proliferation and tube formation is limited to HUVEC, we also investigated AsA effect on another endothelial cell line isolated from human brain i.e. HBMEC. MTT assay results showed that AsA (5–40 µM) decreases cell viability of HBMEC by 2–88% (p≤0.05–0.001) and 12–90% (p≤0.01–0.001) after 6 and 12 h of treatment respectively (Figure 4A). In this assay, the inhibitory effect of AsA on the viability of HBMEC was maximum at higher dose of 40 µM. AsA treatment also inhibited the VEGF-stimulated growth of HBMEC by 8% (p≤0.01), 19% (p≤0.001), 31% (p≤0.001), 54% (p≤0.05) and 66% (p≤0.01) at 5, 10, 15, 20 and 40 µM doses respectively (Figure 4B).

Further, we examined AsA effect on VEGF-stimulated tube formation by HBMEC using the standard two-dimensional matrigel assay. As shown in figure 4C, HBMEC plated on matrigel formed a massive network of tubes (i.e. DMSO treated controls) and thickness of the tubes formed by HBMEC was much stronger than the HUVEC. However, AsA treatment disrupted VEGF-stimulated tubular network formation by HBMEC in a dose-dependent manner (Figure 4C). Tube length measurement revealed that AsA treatment inhibits VEGF-stimulated tube length approximately by 39% (p≤0.001), 53% (p≤0.001), 57% (p≤0.001) and 62% (p≤0.001) at 5, 10, 15 and 20 µM doses, respectively (Figure 4C). These results suggest that the inhibitory effect of AsA on endothelial is not cell line specific.

### AsA treatment strongly inhibits human glioma cell-induced angiogenesis *in vitro*

**Figure 5:**
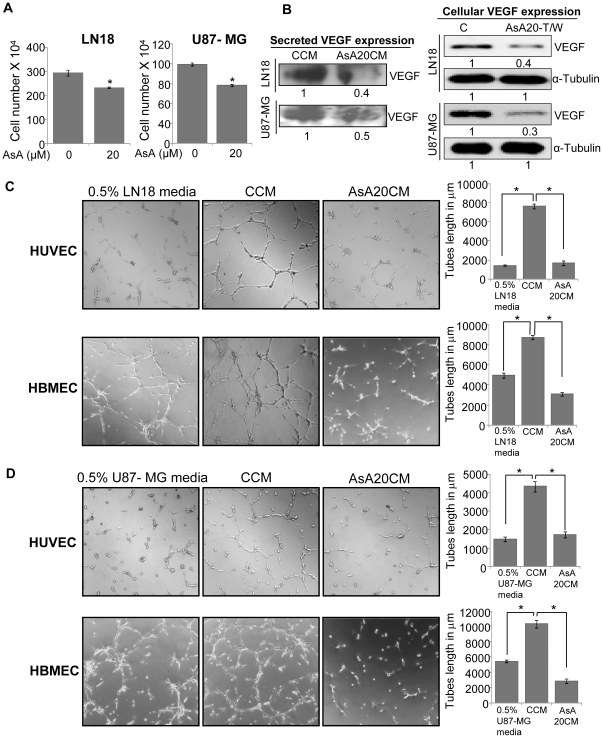
AsA inhibits human glioma cell-induced angiogenesis *in vitro.* **A.** LN18 and U87-MG human glioma cells were treated with AsA at 20 µM doses for 24 h, media was removed, washed with 0.5% serum media and incubated for an additional 12 h in 0.5% serum media without the presence of DMSO or AsA. Subsequently, both LN18 and U87-MG cells were trypsinized, and counted using haemocytometer as detailed in ‘Materials and Methods’. **B.** VEGF expression in CCM (control conditioned media) and AsA20CM (AsA20 conditioned media) or total cell lysates collected from LN18 and U87-MG cells (detailed in ‘Materials and Methods’) was analyzed by Western blotting. The loading volume for conditioned media in each case was normalized with respective cell number. Densitometric values presented below the bands are ‘fold change’ compared to respective controls. AsA20-T/W refers to the group, where glioma cells were treated with AsA 20 µM dose for 24 h and then AsA was washed-out and cell lysates were prepared after 12 h. **C & D.** HUVEC or HBMEC (4×10^4^ per well) were seeded in 24-well plates coated with matrigel and treated with CCM or AsA20CM from LN18 cells or U87-MG cells for 10 h and tube formation assay was performed as described in ‘Materials and Methods’. In this experiment, HUVEC or HBMEC incubated with 0.5% serum containing LN18 or U87-MG media served as a negative control. Tubular structures were photographed at 100x magnification and tube length was measured as described in ‘Materials and Methods’. Tube length data is presented as mean ± standard deviation of three samples for each treatment. The volume for CCM/AsA20CM used in tube formation assay was normalized with respective cell number shown in panel A. *, p≤0.001.

Gliomas are highly vascularized tumors and secrete large amounts of pro-angiogenic factors, which act in a paracrine manner to promote endothelial cell survival and tubular structure formation [12]. Hence, targeting the expression and secretion of pro-angiogenic factors such as VEGF in malignant gliomas could be a promising strategy to inhibit the growth and progression of glioma. Accordingly, we next performed *in vitro* co-culture studies to assess the effect of AsA treatment on the potential of glioma cells to promote angiogenesis. In this study, LN18 and U87-MG human glioma cells were treated with AsA at 20 µM doses for 24 h, thereafter, media was removed and cells were washed with 0.5% serum media and incubated for additional 12 h in 0.5% serum media without the presence of DMSO or AsA. Subsequently, conditioned media was collected, centrifuged and labeled as control conditioned media (from DMSO treated controls, CCM) or AsA conditioned media (from AsA 20 µM treated samples, AsA20CM). After collection of conditioned media, both LN18 and U87-MG cells were trypsinized and counted using haemocytometer and total cell lysate was prepared. As shown in figure 5A, under the described experimental conditions, AsA treatment marginally decreased the live cells number in both the glioma cell lines (LN18 and U87-MG). We preferred such experimental conditions to dissect out anti-angiogenic effects of AsA and to establish that anti-angiogenic effects of AsA are not due to its cytotoxicity towards glioma cells. To fulfill that objective, we also normalized the volume of conditioned media to be used in angiogenesis assay with respective cell numbers from DMSO or AsA treated LN18 and U87-MG cells. Next, we compared the VEGF level in CCM and AsA20CM from both LN18 and U87-MG cells by immunoblotting, and found that AsA20CM from both LN18 and U87-MG cells has lower level of VEGF compared to CCM (Figure 5B, left panel). Under similar experimental conditions, cellular VEGF was also lower in LN18 and U87-MG cells that were earlier exposed to AsA (Figure 5B, right panel). We also observed that CCM from LN18 cells strongly increases tube formation by both HUVEC and HBMEC, while in HUVEC and HBMEC plated with AsA20CM, tube formation was completely diminished both in terms of tubular network quality and tube length (Figure 5C). We observed a similar increase in tube formation by CCM collected from U87-MG cells and here too, the capabilities of HUVEC and HBMEC to form tubular networks in the presence of AsA20CM was significantly reduced (Figure 5D). Whereas the effect of AsA on other angiogenic factors remains unstudied, the results from our completed studies clearly indicate that AsA treatment could inhibit VEGF secretion by LN18 and U87-MG cells; thereby it could inhibit the pro-angiogenic effects of these glioma cells.

### AsA inhibits glioma cell-induced chemotactic motility of endothelial cells

**Figure 6:**
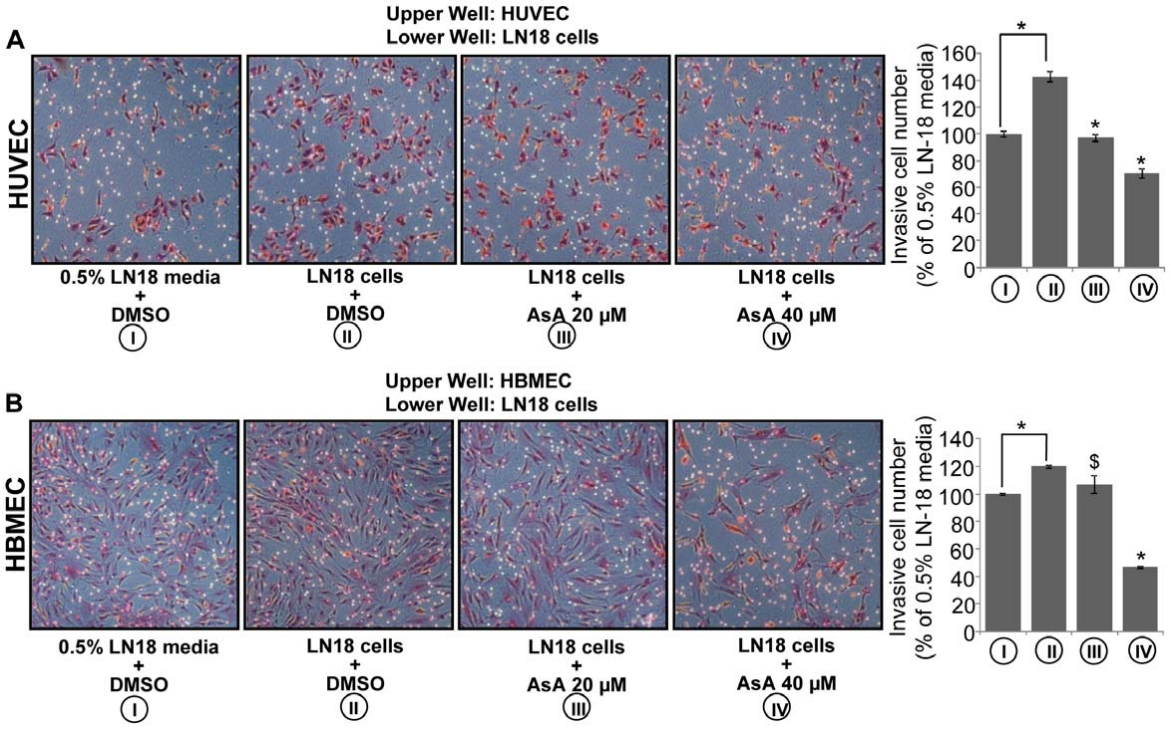
AsA inhibits glioma cells induced chemotactic motility of endothelial cells. **A & B.** HUVEC or HBMEC (3×10^4^ per well) were seeded in the upper chamber of a Transwell plate with 0.5% serum media. In the lower chamber, LN18 cells (3×10^4^ cells per well) were treated with DMSO or AsA 20 µM in 0.5% serum media as described in ‘Materials and Methods’. HUVEC and HBMEC migrated through the matrigel layer were stained and quantified after 10 h and 22 h of their plating in the upper chamber respectively. Cell invasion data shown are mean ± standard deviation of three samples for each treatment. *, p≤0.001; $, p≤0.05.

As mentioned above, glioma cells secrete large amounts of pro-angiogenic factors and attract endothelial cells to enhance neo-angiogenesis [11]. Our analysis of VEGF expression level in the conditioned media of glioma cells suggested that AsA decreases the secretion of VEGF from glioma cells. Hence, in conjunction with this finding, our next aim was to examine whether AsA could inhibit the migration of endothelial cells towards glioma. To study this, we used the transwell migration assay by plating endothelial cells in the upper chamber and glioma cells in the lower chamber, and glioma cells were treated with AsA at 20 and 40 µM doses. Under these experimental conditions, we observed that the presence of LN18 glioma cells in the lower chambers results in higher invasion of HUVEC and HBMEC through matrigel layer compared to only 0.5% serum LN18 media in the lower chamber (Figure 6A & 6B). In the presence of AsA (20 and 40 µM), the potential of glioma cells to attract HUVEC and HBMEC through matrigel layer was significantly compromised. As shown in figure 6A, AsA treatment (20 and 40 µM) of LN18 cells resulted in 33% and 51% reduction in the migration of HUVEC towards LN18 cells. Similarly, AsA treatment (20 and 40 µM) of LN18 cells resulted in 11% and 61% reduction in the migration of HBMEC towards LN18 cells (Figure 6B). Overall, these results clearly showed that AsA treatment inhibits the glioma cell-induced chemotactic motility of endothelial cells.

### AsA inhibits cellular and secreted levels of VEGF in glioma cells

**Figure 7:**
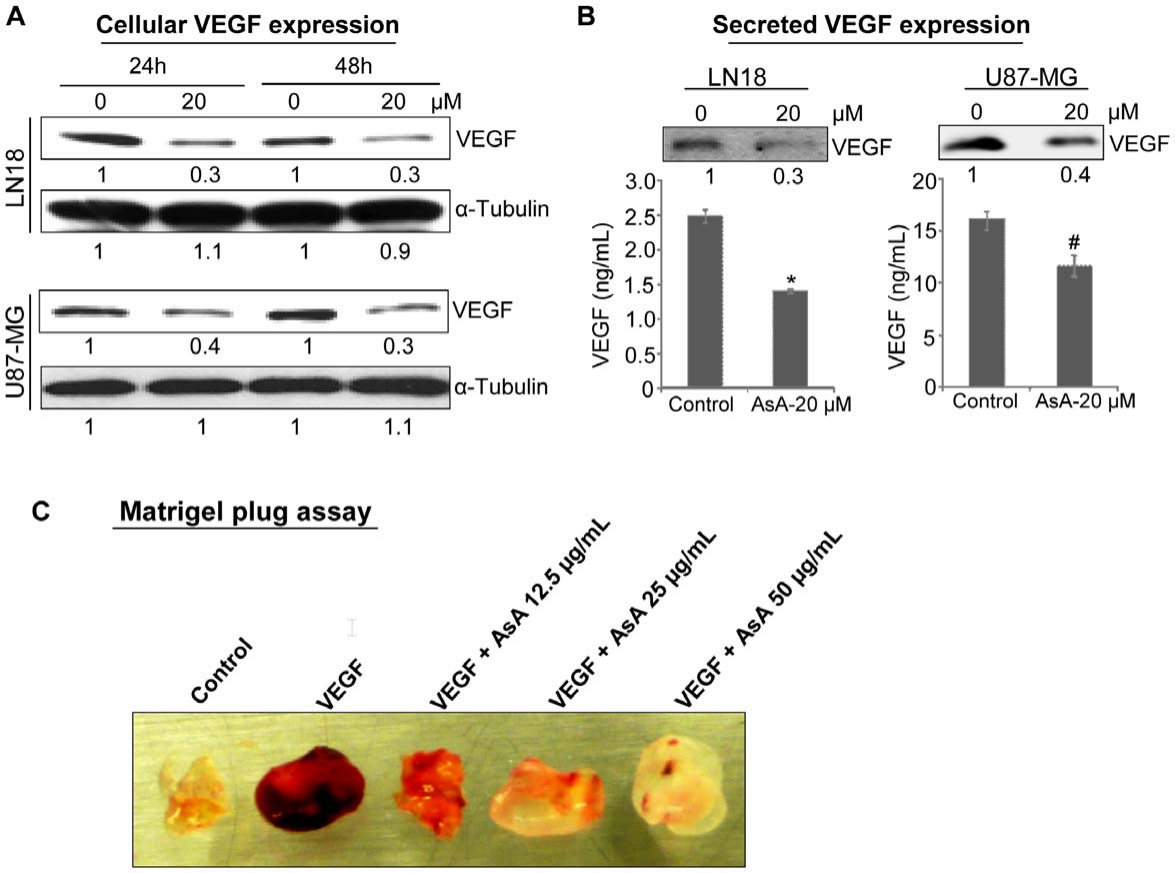
AsA reduces VEGF level (both cellular and secreted) in glioma cells and strongly inhibits VEGF-stimulated angiogenesis *in vivo.* **A.** LN18 and U87-MG cells were treated with DMSO or AsA (20 µM) for 24 and 48 h. After each treatment time, cell lysates were prepared and analyzed for VEGF by Western blotting as described in ‘Materials and Methods’. Membranes were stripped and reprobed with anti- α-tubulin antibody to confirm equal protein loading. Densitometric values presented below the bands are ‘fold change’ compared to respective control after normalization with loading control. **B.** In LN18 and U87-MG cells, media was collected 24 h after AsA treatment (20 µM) and analyzed for secreted VEGF level by Western blotting and ELISA as detailed in ‘Materials and Methods’. **C.** Nude mice were subcutaneously injected with 0.5 mL Matrigel containing 100 ng/mL VEGF, 100 units of heparin and different doses of AsA (12.5, 25 and 50 µg/mL). Matrigel plugs were removed after 5 days and representative pictures are shown. *, p≤0.001; #, p ≤ 0.01.

Next, we examined AsA effect on VEGF level in LN18 and U87-MG cells. As shown in figure 7A, AsA treatment for 24 and 48 h resulted in significant reduction in the VEGF protein level in total cell lysates of both LN18 and U87-MG cells. Since VEGF is actively released by glioma cells, we also examined the effect of AsA treatment on secreted VEGF in the media. ELISA quantitation showed that AsA treatment decreases secreted VEGF level in LN18 and U87-MG cells by 42% (p≤0.001) and 28% (p≤0.01), respectively, which was further confirmed by Western blotting for the secreted VEGF in the media (Figure 7B). Together, these results clearly show that AsA inhibits VEGF levels both cellular and secreted in glioma cells.

### AsA treatment inhibits VEGF-stimulated angiogenesis *in vivo*

To address the *in vivo* anti-angiogenic effect of AsA, we employed the well established Matrigel plug assay. As shown in figure 7C, VEGF presence in the Matrigel plug induced strong vascularity and appeared dark red with blood. The AsA addition in the Matrigel plugs blocked the VEGF-stimulated vascularity in a dose-dependent manner (Figure 7C), supporting the strong anti-angiogenic efficacy of AsA *in vivo*.

## Discussion

Malignant gliomas are the most common brain tumors and are associated with high mortality. Malignant gliomas are resistant to conventional treatment methods and have poor prognosis mainly because of sustained uncontrolled angiogenesis and resultant high tumor mass [11]. It is difficult to remove these tumors surgically without damaging normal nerve tissues. The current radioactive and chemotherapeutic regimens also kill non-specifically many functional and non-cancerous cells in the brain, which might adversely impair different functions of the body. These frightening details warrant immediate alternative strategies to prolong and improve the quality of life of glioma patients. Preventing or inhibiting angiogenesis in glioma using non-toxic and effective phytochemicals could be a useful strategy in this direction. One of the phytochemicals that could be useful against advance glioma is AsA. AsA has a long history of use against numerous neurological disorders with practically no side effects and its use has been shown to improve the physical performance and health-related quality of life [40], [41]. Importantly, oral administration of AsA has been reported to have good plasma availability in humans and there is also sufficient evidence of its reach to brain tissue [30], [40], [41], [42], [43], [44], [45]. In the present work, for the first time, employing *in vitro* co-culture study models we demonstrated the usefulness of AsA against the pro-angiogenic effects of gliomas.

Tumor microenvironment refers to complex cellular and extracellular components surrounding tumor cells at each stage of carcinogenesis [46]. Endothelial cell represent one of the critical cellular elements in the tumor microenvironment that plays a crucial role in the growth and progression of cancer through controlling angiogenesis [46], [47]. Recent literature suggests that anti-angiogenic strategy targeting endothelial cells in the tumor microenvironment could be important as endothelial cells are generally non-transformed and are considered less prone to acquire drug resistance [47], [48], [49]. Therefore, use of nontoxic agents that could effectively target endothelial cells as well as the resultant pathological angiogenesis in the tumor microenvironment could be important in the prevention as well as treatment of cancers. In the present study, AsA treatment strongly inhibited the growth, tube formation as well as invasion/migration in endothelial cells, thereby highlighting its importance as a novel anti-angiogenic agent.

Cell survival is maintained by a delicate balance between anti-apoptotic and pro-apoptotic stimuli. Results from the present study showed that AsA treatment increases the expression of pro-apoptotic molecules (caspase 3, caspase 9 and Bad) while decreasing the expression of pro-survival and anti-apoptotic molecules such as phosphorylated Akt and survivin. Even though the present study was not designed to understand the sequence of signaling events in endothelial cells following AsA treatment, earlier studies have shown that serine/threonine kinase Akt phosphorylates and prevents the pro-apoptotic action of Bad [50]. Furthermore, the role of Akt has been reported in caspases inactivation both directly through phosphorylating caspases and indirectly through promoting the expression of anti-apoptotic molecules such as survivin [50], [51]. More definite studies are still required to understand the mechanism/s underlying AsA-induced apoptosis in endothelial cells.

A large number of pro- and anti-angiogenic cellular factors regulate angiogenesis in gliomas. Among them, VEGF has been implicated as a major paracrine mediator in the pathogenesis of gliomas and it has been shown to directly contribute to angiogenesis and blood brain barrier breakdown [12]. VEGF activates cellular signaling pathways by binding to its receptor tyrosine kinase, which promotes several events required for angiogenesis including endothelial cell survival, proliferation, migration and tube formation [15], [16]. Currently, more than 20 agents targeting VEGF, VEGFR or other members of this signaling cascade have been either approved for cancer treatment or undergoing clinical (phase I–III) studies. In the present study, we found that AsA effectively inhibits VEGF-stimulated proliferation and tube formation in HUVEC and HBMEC. Further, we also observed that AsA inhibits VEGF expression both cellular and secreted in glioma cells; thereby inhibiting pro-angiogenic effects of glioma cells. Further studies are needed to define the effect of AsA on other important anti-angiogenic and pro-angiogenic factors, but these completed studies suggest the important role of VEGF in the anti-angiogenic efficacy of AsA.

In summary, the present study shows that AsA- (a) inhibits HUVEC and HBMEC proliferation, invasion, migration and tube formation; (b) inhibits VEGF-stimulated cell proliferation and tube formation; (c) induces apoptosis by increasing expression of pro-apoptotic signaling molecules, while decreasing the expression of anti-apoptotic signaling molecules; (d) inhibits VEGF expression in glioma cells; and (e) strongly inhibits angiogenesis *in vivo*. In addition, our study provided evidence that AsA could inhibit the glioma cell conditioned media-induced tube formation as well as migration of endothelial cells towards glioma cells by inhibiting the secretion of VEGF from glioma cells. Taken together, our findings suggest that the natural agent AsA could potentially be beneficial as an anti-angiogenic agent and might be a promising chemopreventive agent against gliomas.

## Materials and Methods

### Reagents

Synthetic AsA, with structure confirmed by infrared and proton NMR spectra, was obtained from Sigma-Aldrich (St Louis, MO, USA). The stock solution of AsA was prepared in DMSO and stored at −20°C as small aliquots. Lyophilized recombinant human VEGF was purchased from Invitrogen/Gibco (Camarillo, CA, USA). Matrigel and invasion chambers were purchased from BD Biosciences (New Bedford, MA, USA). Heparin sodium salt was from Tocris Bioscience (Park Ellisville, MO, USA). Primary antibodies for cleaved caspase 3, cleaved caspase 9, cleaved poly(ADP-ribose)polymerase (cPARP), pAkt-ser473, total Akt, Bad, and anti-rabbit peroxidase-conjugated secondary antibody were obtained from Cell Signaling (Beverly, MA, USA). Survivin antibody was from Novus (Littleton, CO, USA). VEGF antibody was from abcam (Cambridge, MA, USA). Human VEGF ELISA kit was from R & D systems (Minneapolis, MN, USA). α-tubulin antibody was from Neomarkers (Fremont, CA, USA). ECL detection system and anti-mouse HRP conjugated secondary antibody were from GE Healthcare (Buckinghamshire, UK). Bio-Rad detergent-compatible protein assay kit was from Bio-Rad Laboratories (Hercules, CA, USA). All other reagents were obtained in their highest purity grade available commercially.

### Cell lines and cell culture

HUVEC were from Lonza (Walkersville, MD, USA) and were cultured in EGM-2 Bulletkit from Lonza (Walkersville, MD, USA) under standard culture conditions (37°C, 95% humidified air and 5% CO_2_). HBMEC were obtained from ScienCell Research Laboratories (Carlsbad, CA, USA) and cultured in endothelial cell media (ScienCell Research Laboratories, Carlsbad, CA, USA). LN18 and U87-MG human glioma cells were from the American Type Culture Collection (ATCC) and cultured in ATCC-formulated Dulbecco's Modified Eagle's Media supplemented with 5% fetal bovine serum (FBS) and Minimum Essential Media, Eagle with Earle's Balanced Salt Solution plus 10% FBS respectively at 37°C under standard culture conditions.

### Cell viability assay

HUVEC (4×10^4^ cells per well) were seeded in complete HUVEC media (EBM-2 basal media containing FBS and growth supplements) in six-well plates. The next day, cells were treated with different doses of AsA (5, 10, 15 and 20 µM) for 6, 12, 24 and 48 h. At the end of each treatment time, total cells were collected by a brief trypsinization and counted using haemocytometer. Trypan blue dye was used for assessing dead cells. In another experiment, HUVEC (4×10^4^ cells per well) were grown under 0.5% serum EBM-2 media and treated with or without VEGF (10 ng/mL) and various doses of AsA for 12 h. At the end of treatment, cell number and cell death were assessed by trypan blue dye using a haemocytometer. To determine cell viability of HBMEC, we used MTT reagent and absorbance was measured by spectrophotometer at 540 nm.

### Quantitative apoptosis assay

AsA effect on apoptosis in HUVEC was quantified by annexin V/PI staining and flow cytometry. Briefly, after AsA treatment (5, 10, 15 and 20 µM for 24 h), cells were collected and washed with PBS twice, and subjected to annexin V and PI staining using Vybrant Apoptosis Assay Kit 2 following the protocol provided by the manufacturer (Invitrogen, Eugene, OR, USA). After staining, flow cytometry was performed for the quantification of apoptotic cells using the flow cytometry core facility at University of Colorado Cancer Center.

### Endothelial cell migration assay

HUVEC were allowed to grow to full confluence in six-well plates. Subsequently, cells were wounded by pipette tips and washed twice with media to remove detached cells, and photomicrographs of initial wounds were taken using Canon Power Shot A640 digital camera (at 100x magnification). Thereafter, cells were treated with DMSO or 5-20 µM doses of AsA. Experiment was terminated as soon as wound was completely filled in DMSO treated controls (after 6 h) and photomicrographs of final wounds were taken for each group. Initial and final wound sizes were measured using AxioVision Rel.4.7 software, and difference between the two was used to determine migration distance using the formula: Initial wound size minus final wound size divided by 2.

### Endothelial cell Transwell migration assay

The chemotactic motility of HUVEC was determined using Transwell migration chambers (BD Biosciences) with 6.5-mm-diameter polycarbonate filters (8-µm pore size) as described previously [47]. In brief, the bottom chambers were filled with 750 µL of EBM-2 media containing all supplements. HUVEC (3×10^4^ per well) were seeded in top chambers with DMSO or various doses of AsA (5, 10 and 15 µM) in 500 µL EBM-2 media with 0.5% serum. Cells were allowed to migrate for 10 h. Non-migrated cells were removed with cotton swabs, and migrated cells were fixed with ice cold methanol and stained with Haematoxylin & Eosin. Images were captured using Cannon Power Shot A640 camera on Zeiss inverted microscope with magnification x100 and invasive cells were quantified by manual counting. In another experiment, we plated LN18 cells in the bottom chamber (DMEM media with 0.5% serum) and treated with either DMSO or AsA. HUVEC or HBMEC were plated in the upper chamber in EBM-2 or ECM media respectively with 0.5% serum and their invasion was studied at defined time points following the protocol detailed above.

### Endothelial cell capillary-like tube formation assay

To examine the effect of AsA on *in vitro* angiogenesis, tube formation assay was performed as described previously [47]. In this assay, we employed two study protocols. In the first protocol, growth factor-reduced matrigel was pipetted into pre-chilled 24-well plates (150 µL matrigel per well) and polymerized for 45 min at 37°C. HUVEC (4×10^4^ per well) in complete media were simultaneously seeded with DMSO or AsA (5, 10, 15 and 20 µM) in matrigel coated plates. After 6 h of incubation, tubular structures were photographed. In the second protocol, DMSO or AsA (5, 10, 15 and 20 µM) treatment was carried out 10 h after HUVEC seeding (when capillary network was already formed). In this case, tubular structures were photographed after 22 h of AsA exposure. Further to check the effect of AsA on VEGF-stimulated tube formation, HUVEC or HBMEC were collected, washed twice with 0.5% serum media and then seeded on matrigel pre-coated plates with or without VEGF (10 ng/mL) along with DMSO or AsA (5, 10, 15 and 20 µM). After 10 h, tubular structures were photographed. In all these experiments, images were captured using Cannon Power Shot A640 camera on Zeiss inverted microscope with magnification x100 and tube length was quantified by AxioVision Rel.4.7 software.

### Conditioned media collection for experimentation

Glioma cells (LN18 and U87-MG cells) were treated with DMSO or 20 µM dose of AsA for 24 h in complete media conditions. Thereafter, media was removed and plates were washed twice with 0.5% serum media and incubated for another 12 h with 0.5% serum media. Subsequently, conditioned media was collected, centrifuged and labeled as control conditioned media (CCM) or AsA20 conditioned media (AsA20CM) and stored at −80°C until further use. For tube formation assay, we optimized a ratio of 75:25 for CCM/AsA20CM and HUVEC/HBMEC media. The glioma cell media with 0.5% serum served as negative control in these experiments.

### Western blotting

To determine the effects of AsA on apoptotic molecules, HUVEC were treated with DMSO or AsA for 24 and 48 h and then whole-cell extracts were prepared in lysis buffer (10 mmol/L Tris-HCl (pH 7.4), 150 mmol/L NaCl, 1% Triton X-100, 1 mmol/L EDTA, 1 mmol/L EGTA, 0.3 mmol/L phenylmethylsulfonyl fluoride, 0.2 mmol/L sodium orthovanadate, 0.5% NP40, and 5 units/mL aprotinin) as described earlier [52]. 60 µg of cellular protein from each sample was denatured in 2X SDS-PAGE sample buffer and resolved on 8–16% Tris-glycine gels. The separated proteins were transferred to a nitrocellulose membrane followed by blocking with 5% nonfat milk powder (w/v) in Tris-buffered saline (10 mM Tris–HCl, pH 7.5, 100 mM NaCl, 0.1% Tween-20) for 1 h at room temperature. After blocking, the membranes were probed with desired primary antibodies for 2 h at room temperature and then overnight at 4°C followed by appropriate peroxidase-conjugated secondary antibody for 1 h at room temperature and visualized by ECL detection system. In each case, blots were subjected to multiple exposures on the film to make sure that the band density is in the linear range. Autoradiograms/bands were scanned and as needed, the mean density of bands was determined using Adobe Photoshop 6.0 (Adobe Systems, Inc., San Jose, CA, USA). To ensure equal protein loading, each membrane was stripped and reprobed with α-tubulin antibody. Wherever applicable, the densitometric value for each band was normalized with respective loading control value i.e. α-tubulin. In another experiment, to determine the effect of AsA on VEGF expression, LN18 and U87-MG cells were treated with AsA at 20 µM for 24 and 48 h and then total-cell extracts were prepared in lysis buffer as described above. In order to assess the effect of AsA on VEGF secretion from glioma cells, media was collected under the above described treatment condition, centrifuged and used for Western blot analysis.

### ELISA assay for VEGF

ELISA assay for quantitative estimation of VEGF in the media from LN18 and U87-MG cells was done as per vendor's protocol (R & D systems).

### *In vivo* Matrigel plug assay

Effect of AsA on VEGF-stimulated angiogenesis *in vivo* was determined by Matrigel plug assay. Briefly, nude mice were subcutaneously injected with 0.5 mL of Matrigel containing 100 ng/mL VEGF, 100 units of heparin and different doses of AsA (12.5, 25 and 50 µg/mL). After 5 days, mice were sacrificed and the skin of each mouse was pulled back to expose the Matrigel plug. Pictures of Matrigel plugs were taken using Canon Power Shot A640 digital camera. For each treatment, there were two matrigel plugs and the experiment was repeated once.

### Statistical analysis

All statistical analyses were carried out with Sigma Stat software version 2.03 (Jandel scientific, San Rafael, CA). Mean and standard deviation were used to describe the quantitative data. One-way ANOVA followed by Tukey's test was used for multiple comparisons and a statistically significant difference was considered at p≤0.05.
